# Supplementary material for: Bacilli in the International Space Station
Source: Microorganisms. 2022 Nov 22;10(12):2309. doi: 10.3390/microorganisms10122309 (PMC9782108; doi:10.3390/microorganisms10122309)
Supplement: Supplementary file 1 [file microorganisms-10-02309-s001.zip › microorganisms-2038027-SI.pdf]

microorganisms

MDPI

Supplementary materials

File S1: List of strains compared by the PATRIC Codon Trees service to construct the phylogenetic tree showed in Figure 2.

1) Geobacillus sp. 12AMOR1
2) Geobacillus kaustophilus HTA426
3) Geobacillus thermoleovorans CCB_US3_UF5
4) Geobacillus sp. Y412MC61
5) Geobacillus thermodenitrificans NG80-2
6) Geobacillus sp. LC300
7) Bacillus cytotoxicus NVH 391-98
8) Geobacillus thermoglucosidans TNO-09.020
9) Bacillus toyonensis BCT-7112
10) Bacillus thuringiensis 97-27
11) Bacillus cereus B4264
12) Bacillus cereus ATCC 4342
13) Bacillus cereus F837/76
14) Bacillus thuringiensis HD682
15) Bacillus cereus strain ISSFR-23F
16) Bacillus thuringiensis str. Al Hakam
17) Bacillus cereus ATCC 14579
18) Bacillus cereus strain JEM-2
19) Bacillus cereus strain ISSFR-3F
20) Bacillus cereus strain NJ-W
21) Bacillus cereus strain ISSFR-9F
22) Bacillus cereus ATCC 10987
23) Bacillus anthracis strain delta Sterne
24) Bacillus sp. JEM-1 strain JEM-1

25) Bacillus sp. S1-R4H1-FB strain S1-R4H1-FB
26) Bacillus anthracis A0157
27) Bacillus mycoides ATCC 6462
28) Bacillus thuringiensis BMB171
29) Bacillus sp. S2-R3J1-FB-BA1 strain S2-R3J1-FB-BA1
30) Bacillus anthracis strain Han
31) Bacillus anthracis strain Cvac02
32) Bacillus cereus Q1
33) Bacillus anthracis str. Ames
34) Bacillus anthracis strain 2000031021
35) Bacillus anthracis str. V770-NP-1R
36) Bacillus anthracis strain Smith 1013
37) Bacillus sp. S1-R1J2-FB strain S1-R1J2-FB
38) Bacillus cereus FM1
39) Bacillus anthracis PAK-1
40) Bacillus cereus 03BB102
41) Bacillus sp. ISSFR-25F strain ISSFR-25F
42) Bacillus weihenstephanensis strain WSBC 10204
43) Bacillus cereus strain FORC_013
44) Bacillus cereus biovar anthracis str. CI
45) Bacillus cereus 03BB87
46) Bacillus sp. S1-R2T1-FB strain S1-R2T1-FB
47) Bacillus bombysepticus str. Wang
48) Bacillus anthracis str. Sterne
49) Bacillus anthracis RA3
50) Bacillus sp. S1-R5C1-FB strain S1-R5C1-FB
51) Bacillus cereus AH187
52) Bacillus anthracis BA1015

53) Bacillus anthracis Ohio ACB
54) Bacillus anthracis str. Turkey32
55) Bacillus cereus 3a
56) Bacillus anthracis str. H9401
57) Bacillus anthracis K3
58) Bacillus anthracis Vollum 1B
59) Bacillus anthracis str. SVA11
60) Bacillus cereus D17
61) Bacillus mycoides strain 219298
62) Bacillus anthracis Pollino
63) Bacillus weihenstephanensis KBAB4
64) Bacillus anthracis 2002013094
65) Bacillus cereus G9842
66) Bacillus anthracis str. A16R
67) Bacillus cereus AH820
68) Bacillus cereus G9241
69) Bacillus anthracis strain BFV
70) Bacillus thuringiensis serovar kurstaki str. HD73
71) Bacillus anthracis strain A1144
72) Bacillus anthracis str. A0248
73) Bacillus anthracis 9080-G
74) Bacillus cereus E33L
75) Bacillus anthracis str. CDC 684
76) Bacillus anthracis 8903-G
77) Bacillus anthracis str. 'Ames Ancestor'
78) Bacillus anthracis 52-G
79) Bacillus thuringiensis strain YGD22-03
80) Bacillus anthracis str. A16

81) Bacillus anthracis strain Stendal
82) Bacillus thuringiensis serovar morrisoni strain BGSC
83) Bacillus thuringiensis strain YWC2-8
84) Bacillus thuringiensis strain HS18-1
85) Bacillus thuringiensis serovar galleriae HD-29
86) Bacillus cereus 03BB108
87) Bacillus thuringiensis serovar indiana strain HD521
88) Bacillus thuringiensis serovar kurstaki str. YBT-1520
89) Bacillus thuringiensis serovar kurstaki HD 1
90) Bacillus thuringiensis serovar kurstaki str. HD-1
91) Bacillus thuringiensis HD-789
92) Bacillus thuringiensis strain YC-10
93) Bacillus thuringiensis serovar thuringiensis str. IS5056
94) Bacillus thuringiensis serovar tolworthi
95) Bacillus thuringiensis Bt407
96) Bacillus thuringiensis HD1002

File S2: List of genes/proteins selected by the PATRIC Codon Trees service to produce Figure 2.

1) Excinuclease ABC subunit C
2) Oligoendopeptidase F
3) Phosphate regulon sensor protein PhoR (SphS) (EC 2.7.13.3)
4) Dihydrolipoamide dehydrogenase of branched-chain alpha-keto acid dehydrogenase (EC 1.8.1.4)
5) tRNA-i(6)A37 methylthiotransferase (EC 2.8.4.3)
6) N-acetylglucosamine-1-phosphate uridyltransferase (EC 2.7.7.23) / Glucosamine-1-phosphate N-acetyltransferase (EC 2.3.1.157)
7) Glycyl-tRNA synthetase (EC 6.1.1.14)
8) Dihydroorotase (EC 3.5.2.3)
9) 16S rRNA (cytosine(967)-C(5))-methyltransferase (EC 2.1.1.176)
10) ADP-ribose pyrophosphatase of COG1058 family (EC 3.6.1.13) / Nicotinamide-nucleotide amidase (EC 3.5.1.42)
11) Dihydrofolate synthase (EC 6.3.2.12) @ Folylpolyglutamate synthase (EC 6.3.2.17)
12) Cystathionine gamma-synthase (EC 2.5.1.48) @ O-acetylhomoserine sulfhydrylase (EC 2.5.1.49)
13) GTP-binding protein YqeH, required for biogenesis of 30S ribosome subunit
14) Uncharacterized MFS-type transporter YfkF
15) N-acetyl-L,L-diaminopimelate deacetylase (EC 3.5.1.47)
16) Phospho-N-acetylmuramoyl-pentapeptide-transferase (EC 2.7.8.13)
17) Tetraprenyl-beta-curcumene synthase (EC 4.2.3.130)
18) Heat-inducible transcription repressor HrcA
19) ABC transporter, permease protein EscB
20) UPF0348 protein family
21) Ribosomal protein L11 methyltransferase
22) DNA polymerase III delta subunit (EC 2.7.7.7)
23) Dipicolinate synthase subunit A
24) Allergen V5/Tpx-1 related
25) Stage 0 sporulation protein YaaT
26) (2E,6E)-farnesyl diphosphate synthase (EC 2.5.1.10)
27) Uncharacterized membrane protein YkvI

28) UPF0701 protein YicC
29) Prephenate dehydratase (EC 4.2.1.51)
30) FIG00003568: ACT domain protein
31) 2',3'-cyclic-nucleotide 2'-phosphodiesterase, Bsub YmdB
32) Succinate dehydrogenase iron-sulfur protein (EC 1.3.5.1)
33) 16S rRNA (cytidine(1402)-2'-O)-methyltransferase (EC 2.1.1.198)
34) NAD kinase (EC 2.7.1.23)
35) Chromosome (plasmid) partitioning protein ParB
36) 3'-to-5' oligoribonuclease A, Bacillus type
37) HesA/MoeB/ThiF family protein
38) Efflux ABC transporter, permease protein
39) FIG007013: polysaccharide deacetylase, putative
40) NAD kinase (EC 2.7.1.23) homolog
41) RNA polymerase sporulation specific sigma factor SigG
42) Late competence protein ComER, proline oxidase (EC 1.5.1.2)
43) 2,4-dienoyl-CoA reductase (EC 1.3.1.34)
44) Quorum-quenching lactonase YtnP
45) Ribonuclease III (EC 3.1.26.3)
46) Phosphatidylserine decarboxylase (EC 4.1.1.65)
47) Menaquinone-cytochrome C oxidoreductase, cytochrome C subunit
48) Protein-glutamine gamma-glutamyltransferase (EC 2.3.2.13)
49) Bis(5'-nucleosyl)-tetraphosphatase (asymmetrical) (EC 3.6.1.17)
50) Uracil phosphoribosyltransferase (EC 2.4.2.9)
51) Chromosome replication initiation protein DnaD
52) KtrCD potassium uptake system, peripheral membrane component KtrC
53) N-acetylglucosaminyl-L-malate N-acetyl hydrolase
54) Peptidoglycan-N-acetylmuramic acid deacetylase PdaA
55) Ribonuclease HII (EC 3.1.26.4)

56) Heptaprenylglyceryl phosphate synthase (EC 2.5.1.n9)
57) Stage IV sporulation protein FA (SpoIVFA)
58) Uncharacterized protein YjaZ
59) Endonuclease III (EC 4.2.99.18)
60) SSU ribosomal protein S4p (S9e) @ SSU ribosomal protein S4p (S9e), zinc-independent
61) Uracil-DNA glycosylase, family 1 (EC 3.2.2.27)
62) Uncharacterized membrane protein YjbE
63) Stage II sporulation protein related to metaloproteases (SpoIIQ)
64) Peptidyl-tRNA hydrolase (EC 3.1.1.29)
65) Acyl-CoA:1-acyl-sn-glycerol-3-phosphate acyltransferase (EC 2.3.1.51)
66) Cell division protein FtsQ
67) Uncharacterized protein YpeP
68) Acetyltransferase, GNAT family
69) KinB signaling pathway activation protein
70) Spore cortex biosynthesis protein
71) Transcription factor FapR
72) 16S rRNA (guanine(1207)-N(2))-methyltransferase (EC 2.1.1.172)
73) dCMP deaminase (EC 3.5.4.12) @ Late competence protein ComEB
74) Stage III sporulation protein AF
75) Transcriptional repressor for NAD biosynthesis in gram-positives
76) Outer spore coat protein CotE
77) DUF402 family nucleoside diphosphatase
78) NADPH-dependent 7-cyano-7-deazaguanine reductase (EC 1.7.1.13)
79) Hydrolase (HAD superfamily), YqeK
80) UPF0234 protein Yitk
81) ATP synthase delta chain (EC 3.6.3.14)
82) Adenylate cyclase (EC 4.6.1.1)
83) Ribonucleotide reductase transcriptional regulator NrdR

84) 2H phosphoesterase superfamily protein Bsu1186 (yjcG)
85) Peptide-methionine (S)-S-oxide reductase MsrA (EC 1.8.4.11)
86) 16S rRNA (guanine(966)-N(2))-methyltransferase (EC 2.1.1.171)
87) Uncharacterized protein YpjA
88) Cell division initiation protein DivIVA
89) RNA binding protein, contains ribosomal protein S1 domain
90) Metal-dependent hydrolase YbeY, involved in rRNA and/or ribosome maturation and assembly
91) Uncharacterized protein YisN
92) FIG001553: Hydrolase, HAD subfamily IIIA
93) Transamidase GatB domain protein
94) Menaquinone-cytochrome C reductase iron-sulfur subunit
95) Putative iron-sulfur cluster assembly scaffold protein for SUF system, SufE2
96) LSU ribosomal protein L10p (P0)
97) Ribose-5-phosphate isomerase B (EC 5.3.1.6)
98) Histidine triad (HIT) nucleotide-binding protein, similarity with At5g48545 and yeast YDL125C (HNT1)
99) D-aminoacyl-tRNA deacylase (EC 3.1.1.96)
100) Peroxide stress regulator PerR, FUR family

File S3: List of strains selected to construct the phylogenetic tree (PATRIC Codon Trees service) of the ISS strains, compared with the reference strains of the *Bacillus cereus* group, and showed in Figure 3.

1) Bacillus cereus strain ISSFR-23F
2) Bacillus cereus strain JEM-2
3) Bacillus cereus strain ISSFR-3F
4) Bacillus cereus strain ISSFR-9F
5) Bacillus cereus ATCC 14579
6) Bacillus sp. JEM-1 strain JEM-1
7) Bacillus sp. S1-R4H1-FB strain S1-R4H1-FB
8) Bacillus sp. S2-R3J1-FB-BA1 strain S2-R3J1-FB-BA1
9) Bacillus anthracis str. Ames
10) Bacillus sp. S1-R1J2-FB strain S1-R1J2-FB
11) Bacillus sp. ISSFR-25F strain ISSFR-25F
12) Bacillus cereus biovar anthracis str. CI
13) Bacillus sp. S1-R2T1-FB strain S1-R2T1-FB
14) Bacillus sp. S1-R5C1-FB strain S1-R5C1-FB
15) Bacillus thuringiensis serovar kurstaki str. HD-1

File S4: List of genes/proteins selected by the PATRIC Codon Trees service to produce Figure 3.

1) Sensor histidine kinase/response regulator
2) Acetoacetyl-CoA synthetase [leucine] (EC 6.2.1.16)
3) membrane-anchoring domain / Glycerophosphoryl diester phosphodiesterase (EC 3.1.4.46)
4) Two-component sensor kinase SA14-24
5) Oligoendopeptidase F
6) CoA-disulfide reductase (EC 1.8.1.14) / Polysulfide binding and transferase domain
7) Argininosuccinate lyase-like protein
8) Glucan 1,4-alpha-maltohexaosidase (EC 3.2.1.98)
9) Endospore coat-associated protein YheD
10) Deoxyribodipyrimidine photolyase (EC 4.1.99.3)
11) Bis-ABC ATPase
12) DEAD-box ATP-dependent RNA helicase CshB (EC 3.6.4.13)
13) ATP-dependent RNA helicase BA2475
14) Spore cortex-lytic enzyme, N-acetylglucosaminidase SleL
15) Two-component system YycFG regulatory protein YycH
16) Niacin transporter NiaP
17) Oxalate/formate antiporter
18) Ornithine aminotransferase (EC 2.6.1.13)
19) Flavohemoglobin / Nitric oxide dioxygenase (EC 1.14.12.17)
20) ABC transporter, permease protein EscB
21) Aminotransferase BA2899
22) putative membrane protein
23) Cystathionine gamma-lyase (EC 4.4.1.1) @ Homocysteine desulfhydrase (EC 4.4.1.2)
24) Branched-chain amino acid dehydrogenase [deaminating] (EC 1.4.1.9)(EC 1.4.1.23)
25) N-acetyl-L,L-diaminopimelate deacetylase (EC 3.5.1.47)
26) Spore germination endopeptidase Gpr (EC 3.4.24.78)
27) Membrane metalloprotease

28) Cytochrome d ubiquinol oxidase subunit II (EC 1.10.3.-)
29) FIGfam013899: ABC transporter substrate-binding protein
30) Cystathionine gamma-synthase (EC 2.5.1.48) @ O-acetylhomoserine sulfhydrylase (EC 2.5.1.49)
31) Endospore coat-associated protein YutH
32) ABC transporter, permease protein
33) Oxidoreductase, Gfo/Idh/MocA family
34) Zinc ABC transporter, substrate-binding protein ZnuA
35) hypothetical protein
36) Oxidoreductase, aldo/keto reductase family
37) Transcriptional regulator in cluster with unspecified monosaccharide ABC transport system
38) Molybdenum cofactor biosynthesis enzyme and related Fe-S oxidoreductases
39) 3-Oxoadipate enol-lactonase, alpha/beta hydrolase fold family [EC:3.1.1.24]
40) Ser/Thr protein phosphatase family protein
41) Membrane protease family protein BA0301
42) Cytochrome d ubiquinol oxidase subunit II (EC 1.10.3.-)
43) Chromosome (plasmid) partitioning protein ParB
44) UPF0750 membrane protein YvjA
45) Quorum-quenching lactonase YtnP
46) Glycine betaine ABC transport system, permease protein OpuAB
47) RNA binding methyltransferase FtsJ like
48) NAD synthetase (EC 6.3.1.5)
49) Serine/threonine protein kinase
50) 2',3'-cyclic-nucleotide 2'-phosphodiesterase, Bsub YmdB
51) Uncharacterized membrane protein YkoY
52) RNA polymerase sporulation specific sigma factor SigG
53) Inositol-1-monophosphatase (EC 3.1.3.25)
54) 2,4-dienoyl-CoA reductase (EC 1.3.1.34)
55) Alkanesulfonate ABC transporter ATP-binding protein SsuB

56) Ribonuclease III (EC 3.1.26.3)
57) RNA polymerase sporulation specific sigma factor SigE
58) 16S rRNA (guanine(527)-N(7))-methyltransferase (EC 2.1.1.170)
59) 5'-methylthioadenosine/S-adenosylhomocysteine nucleosidase related protein BA2564
60) Uncharacterized protein YwmB
61) N-acetylglucosaminyl-L-malate N-acetyl hydrolase
62) ClpCP protease substrate adapter protein MecA
63) SAM-dependent methyltransferase Rv1498c
64) Phosphopantothenoylcysteine synthetase (EC 6.3.2.5)
65) Substrate-specific component ThiT of thiamin ECF transporter
66) Uncharacterized membrane protein YtaF
67) Cell envelope stress response system LiaFSR, response regulator LiaR(VraR)
68) Redox-sensing transcriptional repressor Rex
69) DUF1054 superfamily protein
70) Phosphohydrolase (MutT/nudix family protein)
71) putative secreted protein
72) Hydrolase, haloacid dehalogenase-like family
73) Nucleoside 5-triphosphatase RdgB (dHAPTP, dITP, XTP-specific) (EC 3.6.1.66)
74) Conserved membrane protein in copper uptake, YcnI
75) Uncharacterized protein YhbD
76) hypothetical protein
77) Secreted and spore coat-associated protein 1, similar to biofilm matrix component TasA and to camelysin
78) putative lipoprotein
79) hypothetical protein
80) Possible caffeoyl-CoA O-methyltransferase (EC 2.1.1.104)
81) UPF0398 protein YpsA
82) Uncharacterized membrane protein YuaF
83) ATP synthase delta chain (EC 3.6.3.14)

84) Possible colicin V production protein
85) SAM-dependent methyltransferase
86) DUF402 family nucleoside diphosphatase
87) Putative metal-dependent hydrolase YfiT
88) ThiJ/PfpI family protein YhbO
89) tRNA (cytidine(34)-2'-O)-methyltransferase (EC 2.1.1.207)
90) UPF0302 protein YpiB
91) Cyclic pyranopterin monophosphate synthase (EC 4.6.1.17)
92) Uncharacterized protein YpoC
93) Inosine/xanthosine triphosphatase
94) UPF0478 protein YtxG
95) UPF0756 membrane protein YtwI
96) hypothetical protein
97) Uncharacterized N-acetyltransferase BT9727_3663 (EC 2.3.1.-)
98) Uncharacterized membrane protein YqhR
99) DNA topology modulation protein FlaR
100) LSU ribosomal protein L9p

Table S1. Unique genetic variants, with a predicted high impact on protein product, of the ISS *Bacillus* strain/s compared with *B. anthracis* str. Ames; the data were extracted from the results presented in Figure 5 based on the PATRIC RAST Sequence-based Comparison tool.

Strain(s) name(s)	Unique genetic variants of the strain/s compared with B. anthracis str. Ames	Unique variant with a predicted high impact on protein product	Gene product	Effect of the variant
**Bacillus sp. ISSFR-23F**	51	1	Ferrous iron transporter FeoB	Frameshift variant
**Bacillus sp. ISSFR-9F**	22	2	Efflux ABC transporter, ATP-binding protein	Start lost
			Uncharacterized protein YknW	Frameshift variant
**Bacillus sp. JEM-1**	51	1	Hypothetical protein	Start lost
**Bacillus sp. S2-R3J1-FB-BA1**	127	2	Hypothetical protein	Stop lost
			Hypothetical protein	Start lost
**Bacillus sp. ISSFR-25F**	287	4	Signal transduction histidine kinase CheA	Frameshift variant
			Hypothetical protein	Stop lost
			Hypothetical protein	Frameshift variant
			Hypothetical protein	Frameshift variant
**Bacillus sp. S1-R2T1-FB**	87	2	Hypothetical protein	Stop gained

			Hypothetical protein	Frameshift variant
**Bacillus sp. ISSFR-23F, Bacillus sp. ISSFR-9F**	5	1	MBL-fold metallo-hydrolase superfamily	Start loss
**Bacillus sp. ISSFR-23F, Bacillus sp. JEM-1**	15	1	Flagellin protein FlaA	Frameshift variant
**Bacillus sp. ISSFR-23F, Bacillus sp. ISSFR-9F, Bacillus sp. JEM-1**	16	1	Nutrient germinant receptor inner membrane subunit A (GerKA/GerAA/GerBA)	Frameshift variant
**Bacillus sp. ISSFR-23F, Bacillus sp. ISSFR-9F, Bacillus sp. S2-R3J1-FB-BA1**	3	1	Hypothetical protein	Start loss
**Bacillus sp. ISSFR-23F, Bacillus sp. ISSFR-9F, Bacillus sp. S1-R2T1-FB**	2	1	Efflux ABC transporter, permease protein	Frameshift variant
**Bacillus sp. ISSFR-23F, Bacillus sp. JEM-2, Bacillus sp. ISSFR-9F, Bacillus sp. JEM-1**	6	1	Mobile element protein	Frameshift variant
**Bacillus sp. ISSFR-23F, Bacillus sp. JEM-2, Bacillus sp. JEM-1, Bacillus sp. S1-R4H1-FB**	53	2	FMN reductase, NADPH-dependent	Stop gained
			Uncharacterized protein YknW	Frameshift variant
**Bacillus sp. ISSFR-23F, Bacillus sp. JEM-2, Bacillus sp. ISSFR-9F, Bacillus sp. JEM-1, Bacillus sp. S1-R4H1-FB**	88	1	Hypothetical protein	Frameshift variant
**Bacillus sp. S1-R4H1-FB, Bacillus sp. S2-R3J1-FB-BA1, Bacillus sp. S1-R1J2-FB, Bacillus sp. ISSFR-25, Bacillus sp. S1-R2T1-FB**	1	1	Mobile element protein	Frameshift variant
**Bacillus sp. ISSFR-23F, Bacillus sp. JEM-2, Bacillus sp. ISSFR-9F, Bacillus sp. JEM-1,**	58	2	Uncharacterized protein YpbB	Frameshift variant

**Bacillus sp. S1-R4H1-FB, Bacillus sp. S1-R1J2-FB**			Quaternary ammonium compound-resistance protein	Frameshift variant
**Bacillus sp. ISSFR-23F, Bacillus sp. JEM-2, Bacillus sp. ISSFR-9F, Bacillus sp. JEM-1, Bacillus sp. S1-R4H1-FB, Bacillus sp. S2-R3J1-FB-BA1, Bacillus sp. S1-R1J2-FB**	53	2	Single-stranded DNA-binding protein	Stop gained
			UDP-N-acetylmuramoyl-dipeptide--2,6-diaminopimelate ligase (EC 6.3.2.13)	Frameshift variant
**Bacillus sp. ISSFR-23F, Bacillus sp. JEM-2, Bacillus sp. ISSFR-9F, Bacillus sp. JEM-1, Bacillus sp. S1-R4H1-FB, Bacillus sp. S1-R1J2-FB, Bacillus sp. S1-R2T1-FB**	146	2	2',3'-cyclic-nucleotide 2'-phosphodiesterase (EC 3.1.4.16)	Stop gained
			Wall-associated protein precursor	Frameshift variant
**Bacillus sp. ISSFR-23F, Bacillus sp. JEM-2, Bacillus sp. JEM-1, Bacillus sp. S1-R4H1-FB, Bacillus sp. S2-R3J1-FB-BA1, Bacillus sp. S1-R1J2-FB, Bacillus sp. S1-R2T1-FB**	17	1	Hypothetical protein	Frameshift variant
**Bacillus sp. ISSFR-23F, Bacillus sp. JEM-2, Bacillus sp. ISSFR-9F, Bacillus sp. JEM-1, Bacillus sp. S1-R4H1-FB, Bacillus sp. S2-R3J1-FB-BA1, Bacillus sp. S1-R1J2-FB, Bacillus sp. S1-R2T1-FB**	1047	17	Hypothetical protein	Stop lost
			Hypothetical protein	Frameshift variant
			MBL-fold metallo-hydrolase superfamily	Stop gained
			Hypothetical protein	Stop lost
			Hypothetical protein	Frameshift variant
			Hypothetical protein	Frameshift variant
			Hypothetical protein	Frameshift variant
			Hypothetical protein	Start lost

			Hypothetical protein	Frameshift variant
			Hypothetical protein	Stop gained
			Putative membrane protein	Frameshift variant
			Hypothetical protein	Frameshift variant
			Hypothetical protein	Stop gained
			Phage protein	Stop lost
			Phage protein	Stop gained
			Putative membrane-spanning protein	Frameshift variant
			Hypothetical protein	Frameshift variant
**Bacillus sp. ISSFR-23F, Bacillus sp. JEM-2, Bacillus sp. ISSFR-9F, Bacillus sp. JEM-1, Bacillus sp. S1-R4H1-FB, Bacillus sp. S1-R1J2-FB, Bacillus sp. ISSFR-25, Bacillus sp. S1-R2T1-FB**	118	2	Hypothetical protein	Stop lost
			Hypothetical protein	Start lost

Table S2. Proteins lacking (indicated with "-") at least in one ISS strain compared with *B. anthracis* str. Ames, the data were extracted from the results showed in Fig.5 (based on PATRIC RAST Sequence-based Comparison tool).

**B. *anthracis* str. Ames genome function**	**ISSFR-23F**	**JEM-2**	**ISSFR-9F**	**JEM-1**	**S1-R4H1-FB**	**S2-R3J1-FB-BA1**	**S1-R1J2-FB**	**ISSFR-25F**	**S1-R2T1-FB**
SSU ribosomal protein S12p (S23e)	+	+	+	+	+	+	+	-	+
UDP-glucose 4-epimerase	+	+	+	+	+	+	+	-	+
LPXTG-motif cell wall anchor domain protein	-	-	-	-	-	-	-	-	-
DNA-3-methyladenine glycosylase II	-	-	-	-	-	-	-	-	-
Antirepressor	-	-	-	-	-	-	-	-	-
Reductase or disulfide isomerase in copper uptake, YcnL	.	.	.	.	.	.	.	.	.
Transcriptional regulator, AcrR family	-	-	-	-	-	-	-	-	-
Putative transporter	-	-	-	-	-	-	-	-	-
cell wall-associated protein, putative	-	-	-	-	-	-	-	-	-
Dienelactone hydrolase and related enzymes	-	-	-	-	-	-	-	-	-
BclA protein	+	+	+	-	-	+	+	+	-
SinI protein, antagonist of SinR	+	+	+	+	-	-	-	-	-
collagen-like protein	-	-	-	+	+	-	-	+	-
Uncharacterized metallophosphoesterase YpbG	-	-	-	-	-	-	-	-	-
Azurin	-	+	+	+	+	+	+	+	+
3-methyl-2-oxobutanoate hydroxymethyltransferase	-	+	+	+	+	+	+	+	+
Pantoate--beta-alanine ligase	-	+	+	+	+	+	+	+	+
Aspartate 1-decarboxylase	-	+	+	+	+	+	+	+	+
Glycosyltransferase	-	-	-	-	-	-	-	-	-
TPR/glycosyl transferase domain protein	-	-	-	-	-	-	-	-	-
Glycosyl transferase, group 2 family	-	-	-	-	-	-	-	-	-

Dienelactone hydrolase family protein	-	+	+	+	+	+	+	+	+
Uncharacterized protein YqjZ	-	+	+	+	+	+	+	+	+
DNA-binding protein	-	+	+	+	+	+	+	+	+
putative lipoprotein	-	+	+	+	+	+	+	+	+
MBL-fold metallo-hydrolase superfamily	-	+	+	+	+	+	+	+	+
Beta-lactamase inhibitory protein II	-	-	-	-	-	-	-	-	-
Exosporium protein G	+	+	+	+	+	+	+	-	+
Uncharacterized oxidoreductase	-	-	-	-	-	-	-	-	-
Uncharacterized oxidoreductase	-	-	-	-	-	-	-	-	-
Putative ESAT-secreted protein, BA2187 homolog	-	+	+	+	+	+	+	+	+
Putative ESAT-secreted protein, BA2188 homolog	-	+	+	+	+	+	+	+	+
Putative ESAT-secreted protein, BA2189 homolog	-	+	+	+	+	+	+	+	+
TSPc, tail specific protease	-	-	-	-	-	-	-	-	-
Membrane protein	+	+	+	+	+	+	+	-	+
putative lipoprotein	-	-	-	-	-	-	-	-	-
Bacillus cereus group-specific protein, uncharacterized	-	-	-	-	-	-	-	-	-
SAM-dependent methyltransferase	-	-	-	-	-	-	-	-	-
Uncharacterized methyltransferase YbaJ	-	-	-	-	-	-	-	-	-
Protein distantly related to SAM-dependent methyltransferases	-	+	+	+	+	+	+	+	+
Excinuclease ABC subunit A domain protein	-	+	+	+	+	+	+	-	+
putative lipoprotein	-	-	-	-	-	-	-	-	-
Resolvase	-	-	-	+	-	-	+	+	-
Mobile element protein	-	-	-	-	-	-	-	-	-
Quaternary ammonium compound-resistance protein	-	-	-	-	-	-	-	-	-
intein homing endonuclease-related protein	-	-	-	-	-	-	-	-	-
possible chromosomal replication initiator protein	-	+	+	+	+	+	+	+	+

Exosporium protein C	+	+	+	+	+	+	+	-	+
Uncharacterized protein BA2912	+	+	+	+	+	+	+	-	+
membrane protein, MmpL family	-	-	-	-	-	-	-	-	-
Cupin domain protein in Autoinducer 2 (AI-2) related operon	-	+	+	+	+	+	+	+	+
3-hydroxy-5-phosphonooxypentane-2,4-dione thiolase	-	+	+	+	+	+	+	+	+
Acetyltransferase, GNAT family	-	+	+	+	+	+	+	+	+
putative lipoprotein	-	+	+	+	+	+	+	+	+
Uridine kinase, type 2	-	-	-	-	-	-	-	-	-
putative lipoprotein	-	-	-	-	-	-	-	-	-
putative phosphotransferase	-	-	-	-	-	-	-	-	-
Uncharacterized protein YhjR	-	-	-	-	-	-	-	-	-
Succinoglycan biosynthesis protein	-	-	-	-	-	-	-	-	-
Methylphosphotriester-DNA--protein-cysteine methyltransferase	-	-	-	-	-	-	-	-	-
O-Methyltransferase involved in polyketide biosynthesis	-	+	+	+	+	+	+	+	+
Fic domain protein, HP1159 type	-	-	-	-	-	-	-	-	-
Membrane protein	+	-	-	-	+	-	-	-	-
ABC transporter, fused permease protein	+	-	-	-	-	-	-	+	-
enhancin family protein	-	-	-	-	-	-	-	-	-
2-hydroxymuconate tautomerase-like protein	-	-	-	-	-	-	-	-	-
glycosyl transferase and polysaccharide deacetylase fusion	-	-	-	-	-	-	-	-	-
Hnh endonuclease	-	-	-	-	-	-	-	-	-
Thymidylate synthase ThyX	-	-	-	-	-	-	-	-	-
DNA-cytosine methyltransferase	-	-	-	-	-	-	-	-	-
Replication protein O	-	-	-	-	-	-	-	-	-
Hydrolase, haloacid dehalogenase-like family	-	-	-	-	-	-	-	-	-
Transcriptional regulator, Xre family	-	-	-	-	-	-	-	-	-

Deoxyuridine 5'-triphosphate nucleotidohydrolase	-	-	-	-	-	-	-	-	-
HlyC domain protein	-	-	+	+	-	-	-	+	-
Membrane protein	-	-	-	-	-	-	-	-	-
Putative dipeptidase YtjP	-	-	-	-	-	-	-	-	-
Phosphoglycerol transferase	-	-	-	-	-	-	-	-	-
Wall-associated protein precursor	-	-	-	-	-	-	-	-	-
HNH homing endonuclease	-	-	-	-	-	-	-	-	-
Glycosyl transferase, group 1 family protein	-	-	-	-	-	-	-	-	-
putative lipoprotein	-	-	-	-	-	-	-	-	-
Collagen adhesion protein	+	-	-	-	-	-	-	-	-
Collagen adhesion protein	+	-	-	+	+	+	-	+	+
Collagen adhesion protein	-	-	-	-	-	-	-	-	-
